# Measuring the impact of migraine for evaluating outcomes of preventive treatments for migraine headaches

**DOI:** 10.1186/s12955-016-0542-3

**Published:** 2016-10-06

**Authors:** Sally Mannix, Anne Skalicky, Dawn C. Buse, Pooja Desai, Sandhya Sapra, Brian Ortmeier, Katherine Widnell, Asha Hareendran

**Affiliations:** 1 Evidera, Bethesda, MD USA; 2 Department of Neurology, Albert Einstein College of Medicine and Montefiore Medical Center, Bronx, NY USA; 3 Amgen Inc, Thousand Oaks, CA USA; 4 Evidera, London, UK

**Keywords:** Migraine, Concept elicitation, Development, Functioning, Instrument, PRO, Headache, COA

## Abstract

**Background:**

Migraine is characterized by headache with symptoms such as intense pain, nausea, vomiting, photophobia, and phonophobia that significantly impact individuals’ lives. The objective of this study was to develop a strategy to measure outcomes from the patients’ perspectives for use in evaluating preventive treatments for migraine.

**Methods:**

This study used a multi-stage process. The first stage included concept identification research through literature review, patient-reported outcome (PRO) instrument content review, and clinician interviews, and resulted in a list of concepts relevant to understand the migraine experience. These results informed the design of the subsequent concept elicitation stage that involved qualitative interviews of adults with migraine to understand their experiences. Information from these two stages was used to develop a conceptual disease model (CDM) of the migraine experience. This CDM was used to identify concepts of interest (COI) to evaluate patient-relevant outcomes for assessing treatment benefit of migraine prophylactics. In the final stage, existing PRO instruments were reviewed to assess coverage of concepts related to the selected COI.

**Results:**

Nine articles from 563 screened abstracts underwent full review to identify migraine-relevant concepts. This concept identification and subsequent concept elicitation interviews (*N =* 32; 21 episodic migraine; 11 chronic migraine) indicated that people with migraine experience difficulties during and between migraine attacks with considerable day-to-day variability in the impact on movement, ability to perform every day and social activities, and emotion. The CDM organized concepts as proximal to and more distal from disease-defining migraine symptoms, and was used to identify *impact on physical function* as the key COI. The item level review of PRO instruments revealed that none of the existing PRO instruments were suitable to collect data on impact of migraine on physical functioning, to evaluate treatment benefit.

**Conclusions:**

The impact of migraine includes impairments in functioning during and between migraine attacks that vary considerably on a daily basis. There is a need for novel PRO instruments that reflect patients’ migraine experience to assess treatment benefit of migraine prophylactics. These instruments must evaluate the concepts identified and be able to capture the variability of patients’ experience.

## Background

Migraine is a common and often debilitating neurologic condition characterized by primary recurrent headaches lasting 4 to 72 h with at least two of the following pain characteristics: unilateral, pulsating, moderate or severe intensity, or aggravated by routine physical activity. In addition, migraine attacks are often accompanied by nausea, vomiting, and sensitivity to light (photophobia) and sound (phonophobia) [1]. Based on the International Headache Society (IHS) guidelines, migraine is classified as episodic (EM) or chronic (CM), with CM defined having 15 or more headache days of which at least 8 meet the criteria for a migraine for at least three months (IHS Classification ICHD-III) [1].

Migraine is about three times more common in women than men, affecting roughly 18 % of women and 6 % of men in the United States [2–8]. Migraine prevalence estimates are fairly comparable across the world, with 11.5 % of adults meeting criteria for a migraine on average [9]. Prevalence is highest during an individual’s peak productivity years, between the ages of 25 and 55 [2, 4], where it has significant impact on daily life with substantial functional impairment that include both physical and emotional ramifications [10]. More than half of people with migraine require bed rest to manage their pain [11], leading to work/school absenteeism [12]. Estimates of the burden of migraine suggest that the average impact of migraine on worker productivity is approximately a loss of four workdays per year and 10 days of reduced productivity [13, 14]. In addition to lost wages and productivity due to absenteeism, many people with migraine also experience reduced productivity while at work [13].

In addition to the substantial impairment during attacks (ictal burden), migraine also causes impairment between attacks (interictal burden). When compared with individuals without migraine, migraineurs report reduced health-related quality of life even during pain-free periods [15, 16]. Interictal burden can include anxiety, anticipation of the next attack, and avoidance of activities due to fear of migraine or headache. This can lead to impairment in work or school, impairment in family and social life, difficulty making plans or commitments, and emotional/affective and cognitive distress [17].

Migraine headaches are commonly treated acutely. Acute treatments range from the use of non-specific acute migraine medications including simple analgesics such as nonsteroidal anti-inflammatory drugs (NSAIDs) or acetaminophen for mild to moderate attacks to migraine-specific acute treatments including triptans and ergot-derivatives for moderate to severe attacks [18–20]. Opioids are reserved for patients with moderate to severe pain who do not respond to, or cannot tolerate non-opioid medications [18–20]. Based on published treatment guidelines, roughly 40 % of the migraine population would also benefit from preventive therapy [21]. However, only approximately 12 % of people with migraine receive any preventive therapy due in part to limited efficacy and significant tolerability and safety issues with available preventive therapies, indicating a large unmet medical need for migraine prophylaxis [21]. The aim of prophylactic treatments for migraine, such as antiepileptics, antidepressants, and antihypertensives, is not only to reduce migraine frequency, but also reduce disability and restore the ability to function [22, 23].

To evaluate the benefit of preventive treatments, it is important to examine whether the treatment is associated with a reduction in the impact of migraine on a patient’s life. Thus, guidelines for clinical trials of migraine treatments recommend the inclusion of Patient Reported Outcome (PRO) instruments to support clinical trial endpoints [24]. PRO instruments included in trials should be sensitive to changes resulting from treatment in the context of clinical trials and reflect the experiences of people with the condition, in this case migraine. In order for PRO instruments to be used to evaluate treatment benefit to support label claims, evidence of the relevance of symptoms and impacts on function or quality of life concepts to the target sample is required by the United States Food and Drug Association (FDA) [25]. The European Medicines Agency have defined health-related quality of life (HRQL) as a broad outcome concept that includes, “the patient’s subjective perception of the impact of his disease and its treatment(s) on his daily life, physical, psychological and social functioning and well-being” in the context of evaluating treatment benefit [26].

Although numerous PRO instruments have been used in migraine studies [27], their suitability to evaluate the treatment benefits of preventive migraine treatments has not been explored. Thus this study sought to identify impacts of migraine that are most relevant to those with EM and CM. The objective of this study was to develop a strategy to measure outcomes from the patients’ perspective for use in evaluating preventive treatments for migraine.

## Methods

**Fig. 1:**
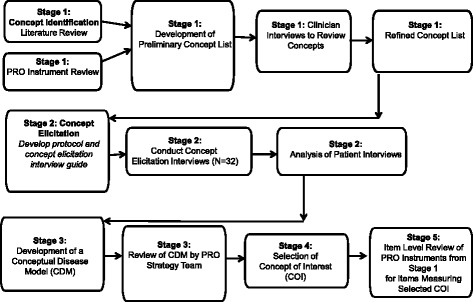
Flow Diagram of the Multi-Stage Project

This study used a multi-stage process. An overview of the flow of the project is illustrated in Fig. 1. The first stage (concept identification through literature review, PRO instrument content review, and clinician interviews) resulted in a list of concepts relevant to understand the experience of people with migraine. These results informed the design of the subsequent concept elicitation stage that involved qualitative interviews with subjects with migraine to understand their experiences. The information from these two stages was used to develop a conceptual disease model (CDM) of the experience of migraine. This CDM was used to identify concepts of interest (COI) to evaluate patient relevant outcomes. Finally, existing PRO instruments were reviewed for coverage of concepts related to the selected the COI to ensure reflection of the patients’ experience of migraine.

The methods and results from each stage of the research are presented in chronological order: concept identification (Stage 1), concept elicitation (Stage 2), development of a CDM (Stage 3), selection of COI (Stage 4), and item level review of the content of PRO instruments to identify best instrument to measure the COI (Stage 5).

## Results and Discussion

### Concept identification (stage 1)

Concept identification research was conducted to understand the impact of migraine in adults. This stage included identifying a list of concepts describing the experience of the impact of migraine symptoms in order to develop a CDM and identify the COI.

#### Concept identification methods

A focused literature review was conducted to identify concepts that reflect patient experiences of migraine. Articles published between 1998 and 2013 reporting qualitative studies related to migraine were identified using key search terms of EMBASE/MEDLINE databases.. Articles selected were reviewed to identify concepts relevant to understand patients’ experience of the impact of migraine. The articles included qualitative studies about individuals’ experience of migraine/headache and its impacts, methodology papers about gathering information on headache/migraine-related impairment, and migraine impact studies.

Next, migraine PRO instruments were identified through a focused search of published articles, including publications on clinical trials and validation studies, through key literature databases (EMBASE/MEDLINE, PsycInfo, and International Pharmaceutical Abstracts) between 2010 and 2012. The PRO instruments identified were reviewed to identify concepts relevant to evaluating the experiences of migraine patients in clinical trials. A preliminary concept list was developed based on the steps above. This list was reviewed by three clinicians in order to provide expert feedback.

#### Concept identification results

In the literature review a total of 563 abstracts were screened. Abstracts related to epidemiological studies, randomized clinical trials, health economic studies, quantitative studies, and development and validation studies were excluded. Nine articles relevant to the objectives of the review were retrieved for full text review [14, 28–35]. The specific impact concepts identified were related to 1) performing day-to-day activities (e.g., at work or school; household chores/tasks); needing to rest, lie down, or sleep), 2) participating in social and leisure activities (e.g., engaging in social activities; being able to participate in leisure activities; maintaining relationships; engaging in sexual activity), 3) emotional responses (e.g., feeling irritable or frustrated; experiencing depressed mood; anxiety, concern or worry in response to migraine), and 4) cognitive difficulties (e.g., ability to concentrate; ability to think clearly).

Twenty-one PRO instruments were reviewed for the concept identification review. These PRO instruments included items to collect data about migraine symptoms as well as impacts of migraine. Common impact concepts included, impact on work-productivity (e.g., mean number of hours/days of work lost and of restricted activity; cost of restricted activity; total rate of lost productivity, in hours, per migraine subject), impact on emotions (e.g., worrying about migraine, anxiety in anticipation of the next migraine, phobic avoidance of activities for fear of migraine headache) and social function (e.g., sexual life, love, friends, social position, leisure time, family situation), and psychological stress (e.g., depression, emotional distress and affective distress).

A preliminary concept list developed based on the steps above was reviewed by three migraine experts. All three migraine experts routinely managed migraine patients in clinic settings, as well as conducted research in migraine. Migraine experts’ feedback was elicited via interviews to ensure clinical relevance, and the concept list was revised to exclude items that were not clinically relevant to understanding patients’ experience of the impact of migraine (e.g., co-morbid conditions were excluded).

### Concept elicitation (stage 2)

The FDA guidance document for industry, “Patient-Reported Outcome Measures: Use in Medical Product Development to Support Labeling Claims” emphasizes the importance of conducting qualitative research throughout the process of instrument development to ensure that the content of the measure is consistent with patients’ experiences and to ensure that the concepts measured by the instrument cover what patients consider most important about their condition [25, 36]. Stage 2 of the research involved the design and conduct of a qualitative concept elicitation study.

#### Concept elicitation methods

A qualitative research protocol, including an interview guide specifically designed to elicit concepts from patients with migraine, was developed based on the results from Stage 1. Concept elicitation interviews were conducted with 32 adult subjects with migraine to explore patients’ experience of the impact of migraine. Adult subjects (18 to 60 years of age) with EM or CM (IHS Classification ICHD-II) with or without aura were recruited through five clinical sites across the United States between August and October 2013. Subjects were recruited using a standard screening script to introduce the study consistently to all subjects. Subjects had to have a history of migraine headache for at least 12 months with at least four headache-free days per month and have experienced a migraine headache within two weeks prior to screening. Key exclusion criteria included migraine onset older than 50 years of age, more than one migraine lasting more than 72 h within three months prior to screening, and cognitive impairment preventing participation in the interview. Subjects with generalized anxiety disorder or major depression were permitted in the study if they were on monotherapy treatment, had not experienced a medication adjustment in the 3 months prior to screening and demonstrated clinical stability. All subjects provided written informed consent prior to data collection procedures. All study procedures were approved by an Institutional Review Board (IRB).

One-on-one in-person interviews were conducted by researchers trained and experienced in qualitative interviewing methods. A semi-structured interview guide was used for the discussions during the concept elicitation interviews (Table 1). The interview guide was designed to elicit concepts about the subjects’ current experiences with migraine, such as the impact of symptoms on functioning. Interviews started with open ended questions for concept elicitation followed by probes on the concepts identified from Stage 1. Each interview was audio-recorded and subsequently professionally transcribed for analysis.

**Table 1:** Outline of the concept elicitation guide

Themes to be explored	Examples of questions
1. History of migraine	Diagnosis, medications, treatments
2. Migraine symptoms	How often? How much intensity/severity)? When?
3. Exploration of symptom impact/impact on functioning migraine has on subject’s life	How is your life impacted by your migraines?
4. Impact migraine symptoms have on subject’s physical function	How is your physical function affected by your migraines?
5. Impact migraine symptoms have on subject’s ability to do day-to-day activities	How is your everyday activity affected by your migraines?
6. Impact migraine symptoms have on subject’s social or recreational activities	How are your social or recreational activities affected by your migraines?
7. Impact migraine symptoms have on subject’s relationships	How is your personal or family life affected by your migraines?
8. Impact migraine symptoms have on subject’s emotions	How are your emotions affected by your migraines?
9. Exploring cognitive impact migraine symptoms have on subject’s life	What cognitive impact from migraines have you noticed in your life?
10. Overall ranking	What aspect of having a migraine has the MOST IMPACT on your life?

#### Qualitative analyses

A saturation grid based on interviewer notes was devised to establish and document saturation as suggested by ISPOR Task force [37]. Saturation is defined as the point at which no substantially new themes, descriptions of a concept, or terms are introduced as additional interviews are conducted [38]. Each subject interview was grouped by sets of five successive subjects to aid with evaluating saturation (i.e., the first five subjects were grouped together, the next five subjects were grouped together, etc.). To ensure that saturation was achieved, structured counts were taken during each interview for the number of interview subjects that endorsed emergent concepts along with review of the coded ATLAS.ti outputs.

Transcript data was imported into ATLAS.ti (version 7.1) qualitative data analysis software (Scientific Software Development GmbH) and analyzed using an open coding approach. Patient statements (e.g., words and phrases) were systematically categorized to identify concepts both distinct to each subject’s experience and common to the experience of migraine. A coding dictionary was then developed based on the concepts that emerged and was expanded as necessary to include emergent concepts that arose during the interviews. The coding dictionary was used to label the patient statements with codes to capture the concepts and themes for analyses.

#### Concept elicitation results

Of the 32 migraineurs (21 EM; 11 CM) interviewed, most (*n =* 27; 84 %) were female and their mean (SD) age was 40.3 (11.3) years (Table 2). Subjects reported experiencing 7.5 (4.1) [mean (SD)] migraine attacks each month. Subjects discussed how the impacts of migraine varied day to day. For example the intensity of the migraine often had a direct and immediate impact on their ability to function during and after the episode. Impacts on physical functioning from migraine symptoms were reported by more than 50 % of subjects. A total of 28 (88 %) subjects spontaneously reported that migraine impacted their physical ability. Impact on physical ability mentioned included - needing to rest or lie down (*n =* 32; 100 %), difficulty with moving one’s head (*n =* 28; 88 %) or body (*n =* 26; 81 %), bending over (*n =* 26; 81 %), walking (*n =* 26; 81 %), getting out of bed (*n =* 26; 81 %), and doing activities requiring physical effort (*n =* 26; 81 %) — especially during the migraine. A total of 25 (78 %) of subjects spontaneously reported that migraine in some way affected their ability to do everyday activities. Impact on everyday activities included missing school or work (*n =* 30; 94 %), difficulty doing daily chores or errands outside the home (*n =* 29; 91 %), difficulty caring for others (*n =* 27; 84 %), being unable to do activities in the presence of loud noises (*n =* 25; 78 %) or bright lights (*n =* 24; 75 %), reduced performance in work/school activities (*n =* 25; 78 %), being unable to do activities requiring concentration (*n =* 25; 78 %) or clear thinking (*n =* 23; 72 %), difficulty with self-care tasks (*n =* 20; 63 %), and an inability to keep a schedule (*n =* 14; 44 %).

**Table 2:** Subject characteristics

	Overall *N =* 32	Episodic migraine *N =* 21	Chronic migraine *N =* 11
Age (years), mean (SD)	40.3 (11.3)	39.7 (11.1)	41.5 (12.1)
Sex (female), *n* (%)	27 (84.4)	17 (81.0)	10 (90.1)
Ethnicity (not Hispanic or Latino), *n* (%)	28 (87.5)	17 (81.0)	11 (100)
Race, *n* (%)^a^			
White	26 (81.3)	17 (81.0)	9 (81.8)
Black or African American	6 (18.8)	3 (14.3)	3 (27.3)
American Indian or Alaska Native	2 (6.3)	1 (4.8)	1 (9.1)
Other	1 (3.1)	0 (0)	1 (9.1)
Employment status, *n* (%)^a^			
Employed, full-time	19 (59.4)	15 (71.4)	4 (36.4)
Employed, part-time	10 (31.3)	5 (23.8)	5 (45.5)
Student	2 (6.3)	2 (9.5)	0 (0)
Unemployed	1 (3.1)	0 (0)	1 (9.1)
Other	1 (3.1)	0 (0)	1 (9.1)
Highest level of education, *n* (%)			
Secondary/high school	3 (9.4)	0 (0.0)	3 (27.3)
Some college	14 (43.8)	10 (47.6)	4 (36.4)
College degree	13 (40.6)	10 (47.6)	3 (27.3)
Postgraduate degree	2 (6.3)	1 (4.8)	1 (9.1)
Migraine diagnosis duration (years), mean (SD)	14.3 (9.7)	12.2 (8.4)	18.5 (11.0)
Migraine interference with daily activities in past week, *n* (%)			
Not at all (0)	1 (3.1)	1 (4.8)	0 (0)
Mildly (1–3)	5 (15.6)	2 (9.5)	3 (27.3)
Moderately (4–6)	14 (43.8)	10 (47.6)	4 (36.4)
Markedly (7–9)	9 (28.1)	6 (28.6)	3 (27.3)
Extremely (10)	3 (9.4)	2 (9.5)	1 (9.1)
Did you miss work or school due to migraine related symptoms in the past week? *n* (%)			
Yes	9 (28.1)	7 (33.3)	2 (18.2)
No	20 (62.5)	14 (66.7)	6 (54.6)
I do not attend work or school	3 (9.4)	0 (0)	3 (27.3)
Treatments taken to treat migraines when they occur, *n* (%)^a^			
Over the counter/non-prescription medication	24 (75)	19 (90.5)	5 (45.6)
Prescription drug	18 (56.3)	10 (47.6)	8 (72.7)

^a^Not mutually exclusive

A total of 22 (69 %) subjects spontaneously reported that migraine affected their social and leisure functioning, including their participation in social events, hobbies, exercise, and activities with family. When probed about the impacts of migraine on social and leisure functioning, 91 % (*n =* 29) endorsed at least one type of impact. Impact on social/leisure functioning included impacts on spending time with family (*n =* 29; 91 %), limiting social interactions (*n =* 25; 78 %), impact on hobbies (*n =* 24; 75 %), impact on partner/spouse relationships (*n =* 20; 63 %), impact on traveling and vacationing (*n =* 20; 63 %), and impact on social activities around loud noises (*n =* 14; 44 %) or bright lights (*n =* 13; 41 %).

Over half of the subjects spontaneously reported the emotional impact of their migraine (*n =* 19; 59 %). When probed about the emotional impacts of migraine, 97 % (*n =* 31) endorsed at least one type of emotional impact. Emotional impact included feeling frustrated or irritated (*n =* 31; 97 %), feeling like a disruption to others when migraine attacks occur (*n =* 29; 91 %), feeling like a burden to others (*n =* 27; 84 %), feeling disappointed or discouraged (*n =* 26; 81 %), worrying (*n =* 27, 84 %), feeling a lack of control (*n =* 20; 63 %), an impact on the ability to show affection (*n =* 16; 50 %), and feeling embarrassed over impairments (*n =* 13; 41 %).

Subjects with EM and CM experienced wide variability in the frequency, duration, and severity of their migraines; however, the same impacts of migraines on physical, social, emotional function, and were described by both CM and EM subjects.

**Fig. 2:**
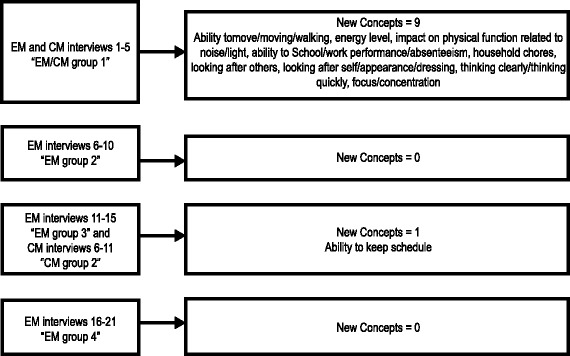
Evidence of Saturation for Physical Functioning

Saturation was reached for EM participants after 15 interviews and for CM participants after 10 interviews. An example of evidence of saturation for physical functioning is shown in Fig. 2. Table 3 presents a selection of subject quotes for the key themes related to the impact of migraine.

**Table 3:** Example quotes by domain

Domain	Episodic migraine quote	Chronic migraine quote
Physical functioning	*You just don’t move around as much…and you don’t move real fast, you don’t want to dance, you don’t want to do anything like that that will kind of jolt your body. (EM no aura*)	*I don’t like to do anything when I’m in the middle of a migraine. I mean, even just any slight movement hurts. I don’t want to get up and go to the bathroom, I don’t. (CM no aura)*
Social and leisure functioning	*Okay. So I’ve had to skip weddings, I’ve had to skip school events, I’ve had to skip my, uh, grandchild’s, um, baptism I had to skip because I had a migraine. Um, just activities, you know, just normal everyday life things that you look forward to. You know, I haven’t been to gone—do because, you know, migraine and, um, you know, and I push myself to try to go to this stuff and, you know, and there’s—then I start throwing up and it’s like, no, I can’t, you know, so. (EM no aura)*	*Well, whether it be visiting, uh, parents to, uh, to recreational sports; whether it be fishing, boating, uh, going to the beach, or the playground…swimming at a neighbor’s house, or going out to eat, any, you know, anything like that. I just wouldn’t do it. (001–006 – CM no aura)*
Emotional functioning	*[Migraines]…just add a lot of stress because of, um, having to worry about like working around it or working through it or making arrangements to be able to not have to do things. (EM aura)*	*I feel like I’m a burden to people when I have migraines, you know, because I have to rely on them and my whole independence is taken away, you know, but, um, when I don’t have a migraine I’m, you know, I’m happy go lucky. (CM no aura)*

### Development conceptual disease model (stage 3)

A conceptual disease model is a pictorial representation of disease processes. A CDM might include information about pathophysiology, natural history, known signs and symptoms, and hypothesized or known outcomes of disease. Development of a CDM is considered a good research practice to help determine which concepts might be helpful to measure using a PRO instrument, in a specific context of use [37].

A CDM was developed based on the results of Stages 1 and 2. The list of concepts identified in Stage 1 and the concepts that endorsed by subjects in Stage 2, were utilized to draft a CDM that visually organized the key concepts which reflect the patients’ experiences of migraine. The CDM was developed to identify and prioritize concepts of interest and to aid in selecting endpoints to evaluate treatment benefits of migraine prophylactics from the patients’ perspective.

**Fig. 3:**
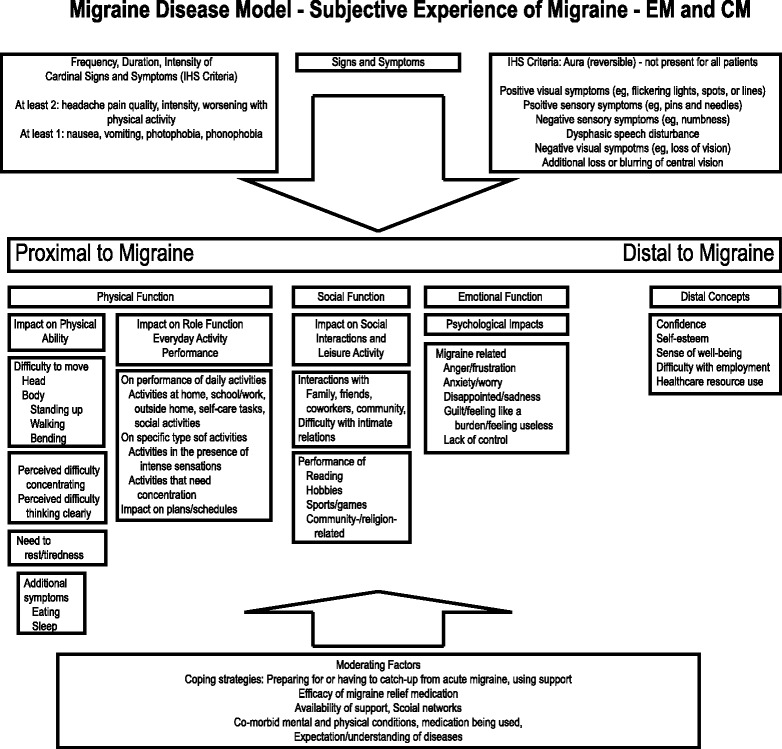
Migraine Disease Model – Subjective Experience of Migraine - EM and CM

The model was further refined based on outputs of structured interviews conducted with two neurologists with expertise in treating migraine patients, a clinical psychologist with expertise in headache and pain management, and an instrument development expert with expertise in migraine-specific PRO tools. Input from the experts was used to revise the model to ensure that the impacts of migraine discussed by the patients were specific to migraine, a result of migraine symptoms (and not comorbid conditions), and were relevant for both EM and CM. Clinicians also indicated it would be helpful for the model to differentiate concepts that were considered disease defining symptoms of migraine (e.g., photophobia) from concepts that could be a result of co-morbid conditions in the target sample (e.g., sleep difficulties, depression). The resulting CDM (Fig. 3) illustrates the key concepts that reflect the patients’ experiences of the disease, and shows how some impacts are more temporally proximal to migraine symptoms.

### Selection of concept of interest (stage 4)

The PRO strategy team used the CDM as a visual aid to help identify the COIs for evaluation of outcomes of prophylactic treatments for migraine. Criteria used to inform the selection of the concept of interest included: at least 50 % of CM and EM subjects needed to experience the impact, consideration of specificity to migraine versus impacts due to additional clinical issues in migraine patients (e.g. comorbid conditions such as depression or anxiety, tolerability issues associated with acute and prophylactic migraine treatments), and aspects related to the context of use of the PRO (e.g., study design aspects like the duration of observation and the potential for the concept to change within that time frame).

The team proposed that the direct impacts of preventing migraines would be experienced in terms of changes in impact on physical functioning. These in turn would also result in changes in the impacts on social functioning and emotional responses to migraine. Impact on physical functioning was therefore selected as the COI that would be most important to evaluate the immediate benefits of interventions that prevented migraines. Evidence of changes in this COI would most likely be observed over a shorter duration of time (approximately 6 months). Concepts temporally distal to migraine symptoms, like the impacts on social and leisure activities and emotional aspects may take a little longer to improve and would be important concepts to assess in the longer term.

### Item level review of content of PRO instruments to evaluate measurement of the COI (stage 5)

An item level review of existing PRO instruments was conducted to determine whether published PRO instruments would be suitable to evaluate the COI selected in Stage 4 - changes in the impact of migraine on physical functioning. Items from each of the existing migraine PRO instruments identified in Stage 1 were reviewed to determine whether they included items that could evaluate the concepts related to physical functioning as reported by the patients in the Stage 2 concept elicitation interviews.

Based on this review, five instruments were identified as potentially relevant for measuring the COI and were the most common migraine PRO instruments identified in clinical studies of migraine therapy: The Migraine Disability Assessment questionnaire (MIDAS) [39]; Migraine-Assessment of Current Therapy (Migraine-ACT) [40]; Headache Impact Test (HIT-6™) [41]; Migraine Specific Quality of life questionnaire (MSQ) [42]; and the Patient Perception of Migraine Questionnaire (PPMQ-R) [43]. However, the PPMQ-R only measured satisfaction with acute migraine treatments and Migraine-ACT was developed only to identify patients requiring a change in their current acute therapy. These two instruments were therefore not considered suitable to evaluate the COI.

The three remaining migraine-specific PRO instruments, Migraine Disability Assessment questionnaire (MIDAS), Migraine Specific QoL questionnaire (MSQ) and the Headache Impact Test (HIT-6), were reviewed in detail and items were mapped to the physical function concepts identified during the concept elicitation interviews (Table 4). In addition, the methods for development and validation of the three identified instruments were reviewed in depth to determine compliance with FDA guidelines for development of PRO instruments to support label claims [11]. While these three instruments included a few items to measure the concepts identified by the subjects in Stage 2 interviews, there was limited evidence to show that they were suitable to comprehensively evaluate the impact of migraine on a physical functioning. For example, none of these instruments included items evaluating difficulty with moving the head [mentioned by 28 patients (88 %) during the CE interviews], difficulty with moving one’s body, getting out of bed, walking, or doing activities requiring physical effort [each mentioned by 26 patients (81 %) during the CE interviews], or difficulty with self-care tasks [mentioned by 20 patients (63 %) during the CE interviews]. Further, none of the instruments had evidence of content validity as proposed in the FDA PRO guidance [25].

**Table 4:** Mapping of concepts covered in the migraine-specific PRO instruments

			Concepts covered			
	Number of items	Recall period	Everyday activity	Impact on movement	Impact on social interactions	Emotional response
Migraine Disability Assessment questionnaire (MIDAS)	5	3 months	On how many days did you miss work or school because of your headaches?			
			How many days was your productivity at work or school reduced by half or more because of your headaches?			
			On how many days did you not do household work because of your headaches?			
			How many days was your productivity in household work reduced by half or more because of your headaches?			
Headache Impact Test (HIT-6)	6	4 weeks	How often does your headache limit your ability to do usual daily activities (household, work, school, social)?	When you have a headache how often do you wish you could lie down?		
			How often have you felt too tired to do work or daily activities because of your headache?	How often have you felt too tired to do work or daily activities because of your headache?		
			How often did your headaches limit your ability to concentrate on work or daily activities?			
Migraine Specific Quality of Life Questionnaire (MSQ; version 2.1)	14	4 weeks	How often does your headache limit your ability to do usual daily activities (household, work, school, social)?	How often have migraines left you too tired to do work or daily activities?		
			How often have you felt too tired to do work or daily activities because of your headache?	How often have migraines limited the number of days you have felt energetic?		
			How often did your headaches limit your ability to concentrate on work or daily activities?			
			How often does your headache limit your ability to do usual daily activities (household, work, school, social)?			

The MSQ was designed to measure three dimensions: (i) Role Function-Restrictive; (ii) Role Function-Preventive; and (iii) Emotional Function in the past four weeks or in the past week. It was not developed based on concept elicitation interviews with patients with migraine [42]; therefore, the conceptual fit of the items within each domain subscale has not been explicitly substantiated. The HIT-6 was designed to measure impact of headaches; it is not migraine-specific and also did not involve patient input during its development [41] it aims to collect data on the impact of headaches have on the ability to function on the job, at school, at home and in social situations in the past 4 weeks. The MIDAS had similar limitations [39] as it was designed to measure the impact of migraine headaches to determine the level of pain and disability caused by migraine in the past 3 months. None of these three instruments were designed to capture the impact of migraine on physical functioning and the day-to-day variability of the experience as reported by patients in the concept elicitation research.

Although the existing instruments are useful in establishing the impact of migraine and its treatment on patients’ quality of life, the lack of complete coverage of the immediate impacts of migraine, the inability to capture the day-to-day variability of ictal and inter-ictal experiences of migraine patients and the lack of evidence of content validity to meet the FDA guidelines of development of PRO tools demonstrates the need for a new instrument to measure the benefit of prophylactic treatment of migraines.

## Conclusion

This study provides a comprehensive assessment of the functional impact of migraine, specifically those of relevance from the individuals’ perspective. Migraine impacts physical functioning, social and leisure activities, and also has emotional impacts. These impacts are experienced during and between migraine attacks and vary considerably day-to-day. This study utilized an iterative and multi-faceted approach to understand patients’ experiences of the impact of migraine. The result of concept identification research and concept elicitation interviews with migraine patients have been used to develop a CDM that can be used as a visual guide to select the COI for developing strategies for measuring outcomes of interventions for migraine, depending on the context of evaluation.

The suitability of existing PRO instruments for measuring outcomes, specifically, the impact on physical functioning, in studies of migraine was evaluated. The review of the existing migraine-relevant PRO tools demonstrated that many of the existing migraine-related PRO tools included a few items measuring the impact of migraine on physical functioning. However, none of these tools comprehensively covered patients’ experiences (reported in the concept elicitation interviews) about the impact of migraine on physical functioning. There was also limited evidence to support the content validity of the instruments for use in evaluating treatment benefit for migraine prophylaxis as per the FDA PRO Guidance [25]. To evaluate outcomes for preventive treatments fora chronic condition with episodic flares, such as migraine, PRO instruments used ideally should be able to measure the variability in the impact of migraine on patients’ ability to function.

The evidence generated thus far strongly supports the need for new PRO instruments to collect data about the impact of migraine on physical functioning for use in evaluating the benefits of preventive treatments for migraine. Such instruments would be useful in evaluating and monitoring outcomes in both clinical trial and clinical practice settings. Suggested next research steps include development of a new PRO instrument for assessing the impact of migraine on functioning incorporating the concepts identified in this study, reflecting the experiences of patients with episodic and chronic migraine.
